# Drug-Tolerant Cancer Cells Show Reduced Tumor-Initiating Capacity: Depletion of CD44^+^ Cells and Evidence for Epigenetic Mechanisms

**DOI:** 10.1371/journal.pone.0024397

**Published:** 2011-09-15

**Authors:** Hong Yan, Xin Chen, Qiuping Zhang, Jichao Qin, Hangwen Li, Can Liu, Tammy Calhoun-Davis, Luis Della Coletta, Jim Klostergaard, Izabela Fokt, Stanislaw Skora, Waldemar Priebe, Yongyi Bi, Dean G. Tang

**Affiliations:** 1 Department of Molecular Carcinogenesis, the University of Texas M.D. Anderson Cancer Center, Smithville, Texas, United States of America; 2 Wuhan University School of Public Health, Wuhan, Hubei, China; 3 Program in Molecular Carcinogenesis, The University of Texas Graduate School of Biomedical Sciences, Houston, Texas, United States of America; 4 Department of Immunology, Wuhan University School of Basic Medical Science, Wuhan, Hubei, China; 5 Department of Molecular and Cellular Oncology, the University of Texas, M.D. Anderson Cancer Center, Houston, Texas, United States of America; 6 Department of Experimental Therapeutics, the University of Texas, M.D. Anderson Cancer Center, Houston, Texas, United States of America; 7 Centers for Cancer Epigenetics, Stem Cell and Developmental Biology, RNA Interference and Non-coding RNAs, and Molecular Carcinogenesis, the University of Texas M.D. Anderson Cancer Center, Houston, Texas, United States of America; University of Dayton, United States of America

## Abstract

Cancer stem cells (CSCs) possess high tumor-initiating capacity and have been reported to be resistant to therapeutics. Vice versa, therapy-resistant cancer cells seem to manifest CSC phenotypes and properties. It has been generally assumed that drug-resistant cancer cells may all be CSCs although the generality of this assumption is unknown. Here, we chronically treated Du145 prostate cancer cells with etoposide, paclitaxel and some experimental drugs (i.e., staurosporine and 2 paclitaxel analogs), which led to populations of drug-tolerant cells (DTCs). Surprisingly, these DTCs, when implanted either subcutaneously or orthotopically into NOD/SCID mice, exhibited much reduced tumorigenicity or were even non-tumorigenic. Drug-tolerant DLD1 colon cancer cells selected by a similar chronic selection protocol also displayed reduced tumorigenicity whereas drug-tolerant UC14 bladder cancer cells demonstrated either increased or decreased tumor-regenerating capacity. Drug-tolerant Du145 cells demonstrated low proliferative and clonogenic potential and were virtually devoid of CD44^+^ cells. Prospective knockdown of CD44 in Du145 cells inhibited cell proliferation and tumor regeneration, whereas restoration of CD44 expression in drug-tolerant Du145 cells increased cell proliferation and partially increased tumorigenicity. Interestingly, drug-tolerant Du145 cells showed both increases and decreases in many “stemness” genes. Finally, evidence was provided that chronic drug exposure generated DTCs via epigenetic mechanisms involving molecules such as CD44 and KDM5A. Our results thus reveal that 1) not all DTCs are necessarily CSCs; 2) conventional chemotherapeutic drugs such as taxol and etoposide may directly target CD44^+^ tumor-initiating cells; and 3) DTCs generated via chronic drug selection involve epigenetic mechanisms.

## Introduction

The cancer stem cell (CSC) concept, that tumors contain stem-like cancer cells, was proposed decades ago and recently revived to explain the cellular heterogeneity in the tumor. One of the most important criteria for defining CSCs is their enhanced ability to regenerate transplantable tumors that histologically recapitulate the phenotypic heterogeneity of the parental tumor [1]. As such, CSCs are often called tumor-initiating cells. CSCs were first identified in leukemia and, since 2003, have been reported for many human solid tumors including glioma [2], Ewing's sarcoma [3], and cancers of the breast [4], [5], colon [6]–[12], pancreas [13], [14], liver [15]–[17], stomach [18], lung [19], [20], head and neck [21], kidney [22], and ovary [23], [24].

Mounting evidence suggests that CSCs may be more resistant to anti-cancer therapeutics, as shown in leukemic [25] and multiple myeloma [26] stem cells. CD133^+^ CSCs increase following radiation and contribute to glioblastoma radioresistance through preferential activation of the DNA damage checkpoint response and an increase in DNA repair capacity [27]. The CD44^+^CD24^lo/−^ breast CSCs are enriched in breast cancer patients who have received adjuvant chemotherapy [28] and more resistant to some chemotherapeutic drugs [29]. In mouse models of mammary tumors, CSCs have also been shown to be refractory to cisplatin treatment [30]. Furthermore, chemoresistant colon cancer cells display CSC phenotypes [31] and CD133^+^ hepatic CSCs are chemoresistant due to preferential activation of the Akt pathway [32]. These new findings highlight potential involvement of CSCs in therapy resistance and in disease recurrence. It has been assumed that drug-resistant cancer cells may all be enriched in CSCs although the general applicability of this assumption remains untested.

Immunohistochemical staining [33], [34], clonogenic assays [35], [36], as well as tumor transplantation experiments [37]–[41] have provided evidence that human prostate cancer (PCa) also contains stem-like cells. Our systematic studies in xenograft models indicate that PCa cells are heterogeneous with respect to their tumor-initiating capacity with the CD44^+^ cell population harboring both quiescent CSCs and fast proliferating tumor progenitors [38], [42]. A fraction of CD44^+^ PCa cells are slow-cycling, can apparently undergo self-renewal, preferentially express ‘stemness’ genes, and possess high tumorigenic and metastatic potentials. CSCs can be further enriched using CD44^+^α2β1^hi^ marker profile [39] and PCa cell holoclones, in which most cells are CD44^+^α2β1^hi^, contain self-renewing tumor-initiating cells [41]. Our recent work shows that Nanog, essential for the self-renewal and pluripotency of ES cells, is enriched in the CD44^+^ PCa cell population and functionally required for tumor development [43]. In fact, inducible Nanog expression is sufficient to endow CSC phenotypic and functional properties and to promote castration-resistant PCa development [44]. A key unanswered question is whether stem-like PCa cells may behave like some other CSCs being resistant to therapeutics or, alternatively, whether drug treatment would enrich PCa-initiating cells. Here we report the unexpected findings that some drug-tolerant cancer cells are much less tumorigenic or even non-tumorigenic. Surprisingly, the drug-tolerant Du145 PCa cell cultures are devoid of CD44^+^ cells, which, at least partially, account for their reduced tumorigenicity.

## Materials and Methods

Animal-related studies have been approved by the M.D Anderson Cancer Center Institutional IACUC committee (ACUF 08-05-08132). All other studies presented herein were the investigator-initiated and did not require approval from other regulatory bodies.

### Cells, reagents, and animals

Du145, PC3, and UC14 cells were obtained from ATCC and cultured in RPMI containing 7% heat-inactivated FBS, 100 µg/ml streptomycin, and 200 U/ml penicillin (Gibco). DLD1 cells were obtained from ATCC and cultured in DMEM containing 7% FBS with antibiotics. Etoposide (VP16) and paclitaxel were purchased from Sigma. Doxorubicin (Dox) and staurosporine (STS) were bought from Biomol. WP1102 and WP1103 were two newly synthesized paclitaxel analogs with substitutions at the 2′-OH (by the Priebe group; details to be published elsewhere). Primary antibodies used in the current study included: rabbit mAb to CD44 (Abcam) for Western blotting (1∶1,000), mouse mAb to CD44 (BD Pharmingen) for immunostaining (1∶500), mouse mAb to ABCG2 (Abcam; 1∶500), rabbit pAb to Bcl-2 (Santa Cruz; 1∶500), mouse mAbs to p21 and p27 (Santa Cruz; 1∶1,1000 for both), rabbit pAb to hTERT (Santa Cruz; 1∶100), rabbit mAb to GAPDH (Cell Signaling; 1∶2,000) and mAb to β-actin (Cell Signaling; 1∶1,000). Secondary antibodies were purchased from GE Healthcare and ECL Plus reagents were from PerkinElmer Inc. NOD/SCID mice were initially purchased from the Jackson Laboratories (Bar Harbor, ME) and the breeding colonies established in our animal facility and maintained in standard conditions according to the institutional guidelines.

### Establishment of drug-tolerant cells (DTCs) and determination of IC_50_ values

Du145, DLD1, and UC14 cells were initially exposed to various drugs, in quadruplicate wells, at a range of concentrations, i.e., 0, 0.1 nM, 1 nM, 10 nM, 50 nM, 0.1 µM, 0.5 µM, 1 µM, 2 µM, 4 µM, and 10 µM. Drugs were replenished every 3 days and cells were treated continuously for 2 weeks. Cell survival and death were closely monitored under an inverted phase-contrast microscope. At the end of a 2-week treatment, ‘optimal’ drug concentrations were determined based on the criterion that drugs showed significant inhibitory effects on cell expansion but did not completely kill the whole population (∼90% cell killing). The entire experiment was repeated once. These experiments led to the determination of optimal concentrations (indicated in the Text). Thereafter, cancer cells were continuously cultured in the medium containing the optimal concentration of drugs for a minimum of 3 months to establish the DTCs, which were designated as Du145-VP16 cells, Du145-Paclitaxel cells, so on and so forth. The DTCs were routinely cultured in the medium containing the optimal concentrations of individual drugs.

To determine the half-maximal concentrations of inhibition (i.e., IC_50_) of parental cancer cells and the DTCs, 2.5–3.0×10^5^ cells were plated in quadruplicate in 24-well plates. After overnight culture, cells were treated with different concentrations of the initial selection drug or non-selecting drugs (to examine potential cross resistance) for 24–48 h. At the end of treatment, viable cell numbers were counted using trypan blue assays and the GraphPad prism 5.0 software was used to analyze data and calculate the IC_50_ values.

### Clonal and BrdU incorporation assays, immunofluorescence, and immunoblotting

Basic procedures for these experiments have been described in our earlier publications [37]–[39], [43]–[45]. To determine total cell numbers, 5,000 cells were plated in triplicate or quadruplicate in 12-well plates and cultured for 10 days, with fresh medium fed every 3 days. At the end, viable cell numbers were counted using trypan blue. For BrdU assays, cells were plated in triplicate on glass coverslips (10,000 cells/coverslip) overnight and then pulsed with 10 µM BrdU for 4 h. At the end, cells were fixed in 4% paraformaldehyde containing 5% sucrose for 10 minutes. Cells were incubated for 20 min in 1% Triton-100 and then denatured, neutralized, blocked, and incubated with monoclonal anti-BrdU antibody (1∶100) for 1 h at 37°C followed by goat anti-mouse IgG-Alexa Fluor 594 (30 min at 37°C). A total of 500–1000 cells were counted per coverslip and two coverslips were counted for each cell type to determine the percentage of proliferating (i.e., BrdU^+^) cells.

For clonal analysis, 100 cells were plated in triplicate in 6-well plates and cultured for 10 days with fresh medium fed every 3 days. At the end, both holoclones and meroclones [41] were enumerated and the results were presented as the cloning efficiency. Paraclones contained large and senescent cells [41] and generally had <20 cells and therefore were not quantified. Immunofluorescence staining of CD44 was performed as described [38], [45]. For Western blotting. parental Du145 and various drug-tolerant Du145 cells were harvested to prepare whole cell lysate in Western blotting analysis of the molecules indicated in the figure panels. In some experiments, Du145-VP16 cells were first treated with various concentrations of trichostatin A (TSA) or 5′-aza-deoxycytidine (Aza) for 72 h.

### Establishment of GFP-tagged drug-tolerant DU145 cells

Briefly, 293FT packaging cells [43] were transfected with pLL3.7-GFP lentiviral vector [43] together with the packaging plasmids using Lipofectamine. Virus-containing medium was collected 48–72 h later, centrifuged at 3,000 rpm, passed through a 0.45 µm filter to remove debris and finally subjected to ultracentrifugation (20,000 rpm×2 h at 4°C). Drug-tolerant DU145 cells were then infected with the virus at MOI (multiplicity of infection) of 20–25.

### Subcutaneous (s.c) and orthotopic tumor experiments

Basic procedures were previously described [37]–[39], [41], [43], [46]. Briefly, parental and drug-tolerant Du145 (and other) cells at different numbers were injected in 50% Matrigel s.c into the flanks of NOD/SCID mice. When the largest tumor(s) in any group must be terminated by IACUC regulations or the tumor-bearing animals became moribund, all animals in that group were sacrificed and tumors harvested. For orthotopic implantation, animals were anesthetized and cells were injected in a 20-µl medium-Matrigel mixture (1∶1) into the dorsal prostate. When tumor burden became obvious (by palpation), the experiment was terminated, animals sacrificed, and primary tumors together with several organs (i.e. lung, liver, spleen, pancreas, kidney, etc) were dissected and examined for micro and macrometastasis (i.e., GFP^+^ foci) under a Nikon epifluorescence microdissection microscope.

### CD44 knockdown experiments

shRNA-mediated knockdown was performed as recently described [43], [46]. Briefly, 293FT packaging cells were transfected with either pGIPz CD44-shRNA lentiviral vector or pGIPz-NS control vector. The virus-containing culture medium was collected 72 h post transfection, centrifuged at 3,000 rpm, filtered through a 0.45 µm-syringe filter, and finally subjected to ultracentrifugation (20,000 rpm×2 h at 4°C). The viral pellet was reconstituted in the OPTI-MEM medium and used to infect HT1080 fibrosarcoma cells to determine the viral titer. Then Du145 cells were infected with the pGIPz-NS or pGIPz-CD44shRNA viruses at an MOI of 20, and, 24–48 h later, were used in either in vitro characterizations or in vivo tumor experiments.

### CD44 overexpression experiments

The basic retroviral procedure was previously described [45]. Briefly, retroviral vectors, including control vector pBabe-GFP and pBabe-CD44 (Addgene, Cambridge, MA) were transfected into the Ampho-Phoenix 293 cells (ATCC). 48–72 h post transfection, virus-containing culture medium was collected, centrifuged at 3,000 rpm, filtered through a 0.45 µm-syringe filter, and finally subjected to ultracentrifugation (22,000 rpm×2 h at 4°C). The viral pellet was reconstituted in the OPTI-MEM medium and used to infect drug-tolerant Du145 cells for 24–48 h, which were then used in both in vitro and in vivo experiments.

### Therapeutic treatment of orthotopic PC3 tumors with paclitaxel

The basic procedure for therapeutic experiments was recently described [46]. Briefly, PC3-GFP cells were implanted in the dorsal prostate (DP) of male NOD/SCID mice (500,000 cells/DP; n = 10). Three weeks later, 5 animals per group were injected, intravenously, with 15 mg/kg body weight of paclitaxel or vehicle control (PBS). The injections were repeated every week for two more weeks (i.e., a total of 3 injections) and animals were terminated 49 days after tumor cell implantation. The DP tumors were dissected out, imaged, and weighed whereas several organs, including the lung, pancreas, lymph node, liver, and brain, were examined for metastasis on a dissecting epifluorescence microscope [46].

### Analysis of ‘stemness’ gene expression profiles by quantitative reverse transcriptase – polymerase chain reaction (qPCR)

The basic procedure for qPCR analysis was recently described [44], [46]. Briefly, total RNA was extracted from Du145 and Du145-VP16 cells using an RNeasy RNA-purification kit (Qiagen, Valencia, CA). The ABI High-Capacity cDNA Archive Kit (Applied Biosystems, Carlsbad, CA) and random hexamers were used for cDNA synthesis. qPCR was performed by the M.D. Anderson Science-Park Molecular Biology Core Facility using an ABI Prism 7900HT (Applied Biosystems). File Builder 3.1 software (Applied Biosystems) was used to design PCR primers and probes. Human gene-specific primer pairs were used for expression profiling by the SYBR® Green method. The experimental Ct (cycle threshold) was calibrated against that of 18S control product. All amplifications were performed in triplicate. The DDCt method was used to determine the amount of gene product relative to that expressed in parental Du145 cells (1-fold, 100%).

### Statistical analyses

GraphPad prism 5.0 software and F-test were used to compare the IC_50_ values. Unpaired *t*-test was used to compare differences in cell numbers, BrdU^+^% cells, cloning efficiency, CD44^+^% cells, and tumor weights. Fisher's Exact Test was used to compare incidence and latency.

## Results

### Chronic sublethal drug treatment led to drug-tolerant cancer cells

Clinically, many cancer patients are often treated CHRONICALLY by anti-cancer therapeutics. The best example perhaps is CML (chronic myelogenous leukemia) patients who must take imatinib (Gleevec) continuously for years [47]. Furthermore, metronomic chemotherapy – a form of chemo administration characterized by frequent, often daily, extended administration of small doses of conventional chemodrugs without major breaks [48], is emerging as a standard therapeutic regimen for many cancers. Based on the assumptions that CSCs may have a special advantage of surviving therapeutics and are likely the cells that mediate drug resistance, we tested whether cancer cells that have survived CHRONIC drug treatment may all possess CSC properties. We first treated Du145 prostate cancer (PCa) cells with two clinical drugs, i.e., etoposide (VP16) and paclitaxel (Taxol) as well as three experimental drugs, staurosporine (STS), a promiscuous protein kinase inhibitor, and two newly synthesized paclitaxel analogs termed WP1102 and WP1103 (details to be presented elsewhere). As described in Methods, we first treated Du145 cells with these five drugs at a range of 10 concentrations for 2 weeks to determine the ‘optimal’ sublethal concentrations at which drugs significantly inhibited tumor cell expansion but did not kill the entire population. Using this chronic treatment protocol that ‘mimics’ metronomic treatment in the clinic, we determined the optimal concentrations, in Du145 cells, of VP16, paclitaxel, STS, WP1102, and WP1103 at 1.25 µM, 50 nM, 7 nM, 5 nM, and 25 nM, respectively. Du145 cells were subsequently cultured, continuously, in the medium containing the optimal concentrations of drugs for ∼3 months. The resultant drug-tolerant cell (DTC) lines were designated as Du145-VP16, Du145-Paclitaxel, Du145-STS, Du145-WP1102, and Du145-WP1103 cells, respectively.

**Figure 1:**
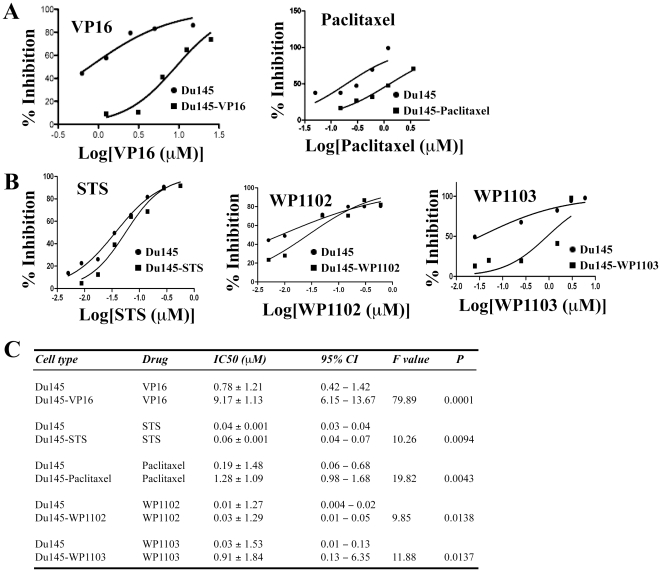
Drug-tolerant Du145 cells were more resistant to the selection drugs. A–B. Parental Du145 and drug-tolerant Du145 cell lines selected with two clinical drugs (A) or three experimental drugs (B) were exposed, side-by-side, to the respective selection drugs (e.g., Du145-VP16 cells to VP16 and Du145-Paclitaxel cells to paclitaxel) at the concentrations (transformed into logarithm) indicated. Y-axis represents cell growth inhibition (%). C. Tabulated presentations of IC_50_ values (see Materials & Methods) and corresponding 95% CI (confidence interval) of Du145 cells and drugs-tolerant Du145 cells in response to the five drugs indicated. Values were calculated from data obtained in A and B.

To determine whether drug-tolerant Du145 cells were truly tolerant of the original selection drugs, we treated parental and drug-tolerant Du145 cells side-by-side with the respective five drugs. As shown in Fig. 1, drug-tolerant Du145 cells were generally more resistant than parental Du145 cells to the selection drugs. Thus, Du145-VP16 cells were >10 times more resistant than Du145 cells to VP16 (IC_50_ values being 0.78 µM for Du145 and 9.17 µM for Du145-VP16 cells). Du145-Paclitaxel cells were ∼7 times more resistant to paclitaxel than Du145 cells (Fig. 1, A and C). Similarly, Du145-WP1103 cells were nearly 30 times more resistant to WP1103 than Du145 cells (Fig. 1B–C). Finally, Du145-STS and Du145-WP1102 cells were approximately 1.5 and 3 times more resistant to STS and WP1102, respectively, than unselected Du145 cells (Fig. 1B–C). Hence, the differential drug resistance among the established DTCs ranked Du145-WP1103>Du145-VP16>Du145-Paclitaxel>Du145-STS≈Du145-WP1102.

**Figure 2:**
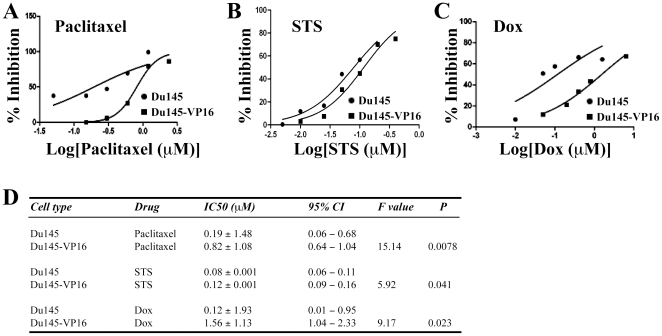
Drug-tolerant Du145 cells were cross-resistant to non-selecting drugs. Du145-VP16 cells were treated with paclitaxel (A), STS (B), and doxorubicin (Dox; C) at the concentrations [log] indicated. Results are presented as % inhibition and IC_50_ (D) were determined as in Figure 1.

Subsequently, we exposed Du145-VP16 cells to three non-selecting drugs, i.e., paclitaxel, STS, and doxorubicin (Dox). As shown in Fig. 2, Du145-VP16 cells were more resistant (than parental Du145 cells) to paclitaxel (∼4 fold), STS (1.5 fold), and Dox (13 fold), suggesting that the DTCs were also cross-resistant to other non-selecting drugs.

**Figure 3:**
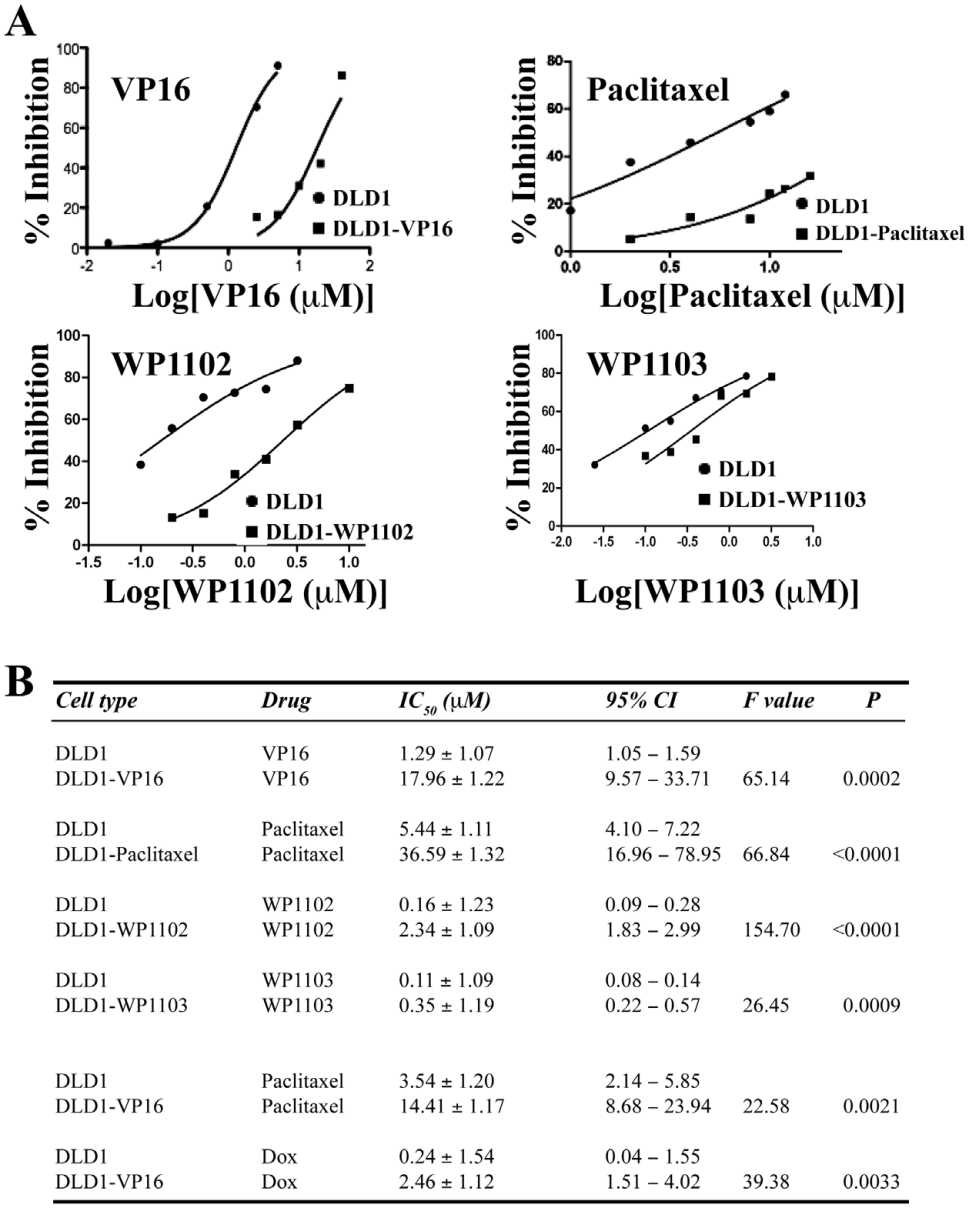
Drug-tolerant DLD1 cells were resistant to selecting and non-selecting drugs. Parental and drug-tolerant DLD1 cell lines selected with optimal concentrations (see Text) of VP16, paclitaxel, WP1102, and WP1103, were exposed, side-by-side, to the respective selection drugs at the concentrations (transformed into logarithm) indicated (A). Y-axis represents cell growth inhibition (%). B. Tabulated presentations of IC_50_ values and corresponding 95% CI (confidence of intervals) of parental DLD1 cells and drug-tolerant DLD1 lines in response to both selecting and non-selecting drugs.

Using similar strategies, we also established drug-tolerant DLD1 colon (Fig. 3) and UC14 bladder (not shown) cancer cells. In DLD1 cells, the optimal concentrations for VP16, paclitaxel, WP1102, and WP1003 were determined to be at 2.5 µM, 100 nM, 120 nM, and 100 nM, respectively. As shown in Fig. 3, the established DLD1-VP16, DLD1-Paclitaxel, DLD1-WP1102, and DLD1-WP1103 cells were resistant to the respective selecting drugs. Furthermore, DLD1-VP16 cells also showed cross resistance to paclitaxel and Dox (Fig. 3B). Drug-tolerant UC14 cells were similarly more resistant to the selection drugs (i.e., paclitaxel, STS, VP16, Dox, WP1102, and WP1103), than the parental UC14 cells (not shown).

### Drug-tolerant Du145 cells were surprisingly less tumorigenic than parental Du145 cells

We hypothesized that the DTCs might possess CSC properties and be more tumorigenic in vivo. Much to our surprise, all drug-tolerant Du145 cells subcutaneously (s.c) injected into the NOD/SCID mice demonstrated much reduced tumor-initiating capacity when compared to the same number of parental Du145 cells, which showed a tumor-initiating frequency (TIF) of ∼1/175 (Table 1). Injection of increasing numbers of parental Du145 cells, expectedly, led to reduced latency (Table 1). In contrast, Du145-VP16 cells showed a TIF of ∼1/62,000 and, at 100,000 cells injected, tumor latency was more than twice as long as for the same number of Du145 cells (Table 1). In fact, both Du145-Paclitaxel and Du145-WP1103 cells were non-tumorigenic up to 10,000 (for Du145-WP1103) and 100,000 (for Du145-Paclitaxel) cells implanted (Table 1). Even for Du145-STS and Du145-WP1102 cells, which showed the lowest IC_50_ differentials (Fig. 1B–C), reduced tumor incidence with TIF of 1/971 and 1/45,787, respectively, and smaller tumors were also observed (Table 1).

**Table 1:** Drug-tolerant Du145 cells possess much reduced tumorigenic potential.

Cell type^a^	Cell#	Incidence (%)^b^	TIF^c^	Latency(d)^d^	Termination(d)^d^	Weight (g)^e^
Du145	100	7/7 (100)	**1/175** (1/66–1/467)	63	90	0.07±0.03 (0.03–0.12)
	1000	7/8 (87.5)		36	60	0.42±0.33 (0.10–0.95)
	10000	8/8 (100)		27	59	0.50±0.35 (0.12–0.97)
	100000	7/8 (87.5)		21	52	0.39±0.17 (0.18–0.65)
Du145-VP16	100	1/8 (12.5)	**1/62,453** (1/26,455–1/147,436)	92	117	1.45
	1000	0/8**			60	
	10000	0/8**			59	
	100000	5/6 (83.3)		51**	52	0.06±0.05 (0.02–0.14)**
Du145-VP16 (2 mo.)#	1000	1/6 (16.7)*	**1/30,110** (1/7,199–1/125,948)	83	127	0.01
	10000	1/6 (16.7)*		45	127	0.2
Du145-STS	100	1/8 (12.5)	**1/971** (1/421–1/2,241)	52*	90	0.16
	1000	5/8 (62.5)		58*	66	0.07±0.02 (0.04–0.09)*
	10000	3/8 (37.5)*		48*	66	0.13±0.14 (0.05–0.29)*
Du145-Paclitaxel	100	0/8**			105	
	1000	0/8**			60	
	10000	0/8**			60	
	100000	0/6**			52	
Du145-WP1102	100	2/4 (50)	**1/45,787** (1/15,313–1/136,901)	58	90	0.03±0.01 (0.02–0.04)*
	1000	2/4 (50)		58**	66	0.37±0.13 (0.27–0.46)
	10000	2/4 (50)		58**	66	0.11±0.12 (0.02–0.19)*
	100000	3/4 (75)		31*	52	0.19±0.24 (0.01–0.46)
Du145-WP1103	100	0/8**			99	
	1000	0/8**			60	
	10000	0/8**			60	
Du145-WP1103 (2 mo.)#	1000	0/6**			108	
	10000	0/6**			108	

a Parental Du145 or drug-tolerant Du145 cells were s.c implanted in 50% Matrigel, at the numbers indicated, in NOD/SCID mice. In the two experiments marked by #, cells were first cultured in drug-free medium for 2 months prior to injections.

b Tumor incidence (% of tumor development/injections).

c Tumor-initiating frequency, as determined using the L-Calc™ software (Stemcell Technologies). The ranges were indicated in the parentheses.

d Tumor latency (mean time in days from injection to when tumors were first palpated) and termination time (days from injection to when animals were sacrificed).

e Mean ± S.D (ranges in parentheses). Note that tumor weights sometimes varied greatly among different cell number groups. For statistical analyses, Fisher's Exact Test was used to compare tumor incidence and Student *t*-test was used to compare tumor weights and latencies.

*P<0.05;

**P<0.01, when compared with the parental Du145 cells of the same number.

**Figure 4:**
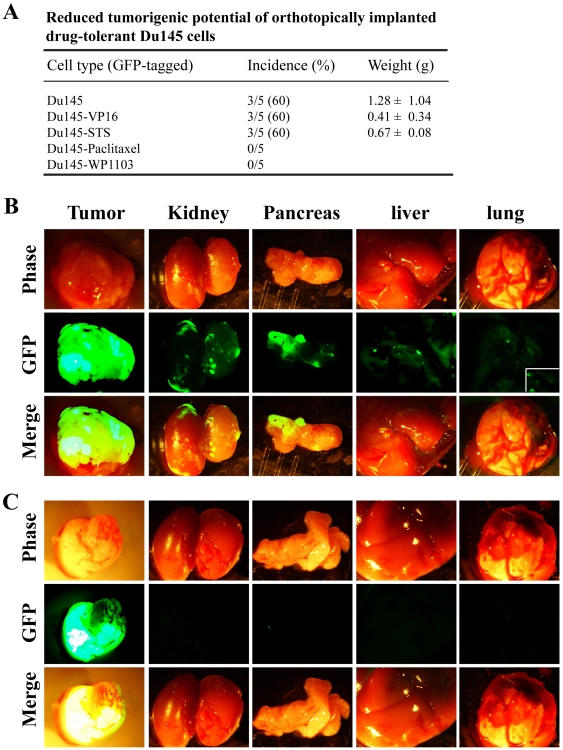
Orthotopically implanted drug-tolerant Du145 cells showed reduced tumorigenic and metastatic potential. A. GFP-tagged parental or drug-tolerant Du145 cells were implanted (500,000 cells/injection), in 50% Matrigel, in the DP of NOD/SCID mice. All animals were terminated 75 days post implantation. Shown are tumor incidence and tumor weights (mean ± S.D; statistics not applicable due to relatively small numbers of animals). B–C. Representative tumor and organ images from a tumor-bearing animal in the Du145 (B) and Du145-VP16 (C) group, respectively. All images were acquired using a Nikon microdissecting epifluorescence microscope at 0.75× and the boxed area in B (the lung GFP image) represents an enlargement showing GFP^+^ spots in the lung.

To determine whether tumor implantation site might have an effect on the differential tumorigenicity observed, we established GFP-tagged Du145 parental and drug-tolerant Du145 cells and implanted equal numbers (i.e., 500,000) of cells orthotopically in the dorsal prostate (DP) of the male NOD/SCID mice. When the experiment was terminated 75 days post tumor cell injections, we observed that Du145-VP16 and Du145-STS cells generated smaller tumors than parental Du145 cells (Fig. 4A). In fact, both Du145-Paclitaxel and Du145-WP1103 cells were non-tumorigenic in the DP tumor regeneration model (Fig. 4A). Significantly, parental Du145 cells metastasized to multiple organs including lymph nodes, kidney, pancreas, liver, lung, and spleen whereas drug-tolerant Du145 cells lacked apparent metastasis to any of these organs (Fig. 4B–C; data not shown).

### Drug-tolerant DLD1 cells also showed reduced tumorigenicity whereas drug-tolerant UC14 cells demonstrated drug-dependent changes in tumor-initiating capacity

To determine whether the reduced tumorigenicity associated with drug-resistant cells is restricted only to Du145 cells, we similarly injected, s.c., increasing numbers of parental DLD1 and four drug-tolerant DLD1 cell lines into the NOD/SCID mice. As shown in Table S1, the drug-tolerant DLD1 cells, though displaying similar tumor incidence, regenerated significantly smaller tumors compared to parental DLD1 cells at the same cell numbers.

We carried out similar limiting-dilution tumor experiments in 6 drug-selected UC14 cells (Table S2). In contrast to drug-tolerant Du145 and DLD1 cells, which uniformly demonstrated reduced tumorigenic potential, 4 of the 6 drug-tolerant UC14 cells (Table S2; shadowed brown) demonstrated enhanced tumor-regeneration capacity compared to the same number of parental UC14 cells. Interestingly, two drug-tolerant UC14 cell lines also regenerated much smaller tumors than the same number of parental UC14 cells (Table S2; shadowed pink). These results indicate that drug-tolerant UC14 cells are either more or less tumorigenic than the parental cells, depending on the initial selection drugs.

### Drug-tolerant Du145 cells were less proliferative and showed low cloning efficiency

**Figure 5:**
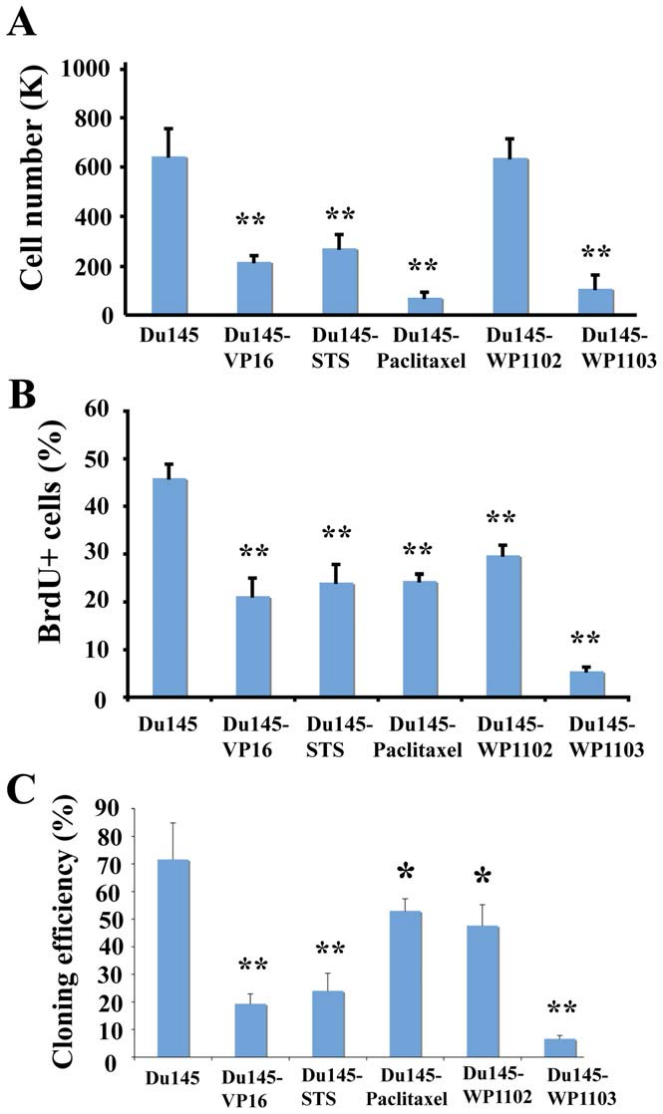
Drug-tolerant Du145 cells were less proliferative and showed low cloning efficiency. A. Quantification of numbers of viable cells. Parent and drug-tolerant Du145 cells were plated, in quadruplicate, in 12-well plates (5,000 cells/well) and viable cells were quantified using Trypan blue exclusion assay 10 days post plating. B. Quantification of % BrdU^+^ cells (see Materials & Methods). C. Determination of cloning efficiency. Parent and drug-tolerant Du145 cells were plated, in quadruplicate, in 6-well plates (100 cells/well) with fresh medium replenished every 3 days. The numbers of holoclones and meroclones were determined 10 days post plating. In all data, bars represent the mean ± S.D and statistical analyses were conducted using Student *t*-test (*, P<0.05; **, P<0.01).

Since it was quite unexpected that some drug-tolerant cancer cells showed reduced tumor-regenerating capacity, we subsequently focused on drug-tolerant Du145 cells in attempt to uncover potential mechanisms. We consistently observed that most drug-tolerant Du145 cells seemed to proliferate more slowly compared to Du145 cells. Indeed, in a prospective 10-day experiment measuring live cell numbers, we observed that all drug-tolerant Du145 cells, except Du145-WP1102 cells, showed much lower end-point live cell numbers (Fig. 5A), suggesting that DTCs were less proliferative and/or more susceptible to cell death. We then carried out BrdU incorporation experiments to directly measure cell proliferation. As shown in Fig. 5B, drug-tolerant Du145 cells exhibited lower proliferative indices (i.e., % BrdU^+^ cells). Interestingly, even Du145-WP1102 cells demonstrated a lower proliferative index (Fig. 5B) although these cultures showed similar total live cell numbers to parental Du145 cells (Fig. 5A). Remember that Du145-WP1102 cells also displayed relatively less reduction in tumor-initiating capacity compared to other drug-tolerant Du145 cells (Table 1). It is possible that Du145-WP1102 cells proliferated less but also had less spontaneous cell death, thus resulting in similar end-point live cell numbers (Fig. 5A) and less pronounced decreases in tumorigenicity (Table 1). Indeed, we consistently observed that Du145-WP1102 cells, chronically selected using 5 nM WP1102 compound, showed less floating and apoptotic cells in the culture flasks compared with Du145-WP1103 cells (not shown), which were chronically selected using 25 nM WP1103 compound. Finally, we observed that all drug-tolerant Du145 cells demonstrated lower cloning efficiencies than the parental Du145 cells (Fig. 5C).

**Figure 6:**
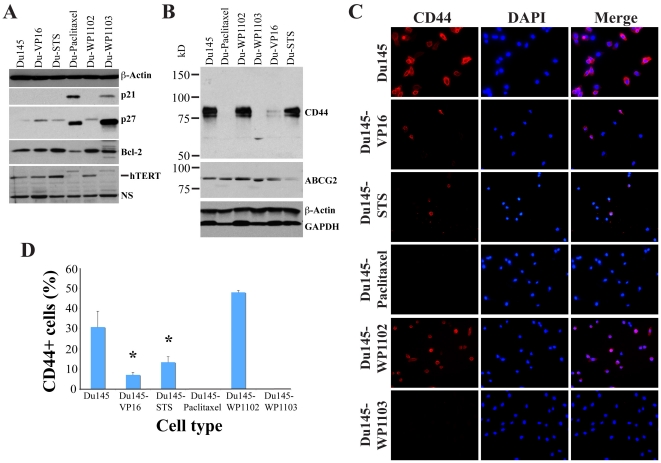
Molecular changes and reduced CD44^+^ cells in drug-tolerant Du145 cell cultures. A. Whole cell lysate from the cell types indicated was used in Western blotting of p21, p27, Bcl-2, and hTERT and the blot was reprobed for β-actin. NS, non-specific. B. Western blotting of CD44 and ABCG2. The blot was reprobed for β-actin and GAPDH. C–D. Du145 and drug-tolerant Du145 cells were plated on glass coverslips and stained for CD44 using monoclonal antibody. Shown are representative images (C) and quantification of CD44^+^ cells (D; mean ± S.D, *, P<0.01).

Consistent with reduced cell proliferation, drug-tolerant Du145 cells showed increased levels of two cyclin-dependent kinase inhibitors, p21 and p27, especially in Du145-Paclitaxel and Du145-WP1103 cells (Fig. 6A), the two cell lines that completely lacked tumorigenicity (Table 1). The p27 levels were also elevated in all three other drug-tolerant Du145 cell lines (Fig. 6A). Intriguingly, the significantly increased p27 protein band in Du145-Paclitaxel and Du145-WP1103 cells migrated slightly faster than the protein in other cell lines (Fig. 6A). Future studies will clarify this potentially interesting observation. In contrast to p21 and p27, Bcl-2, an anti-apoptotic protein, showed less, albeit dramatic decrements, again, in the two non-tumorigenic Du145 lines, i.e., Du145-Paclitaxel and Du145-WP1103 (Fig. 6A).

### Drug-tolerant Du145 cultures showed reduced numbers of, or were devoid of, CD44^+^ cells

Several pieces of evidence suggest that diminished tumor-regenerating capacity in drug-tolerant Du145 cells may involve, in addition to compromised proliferative potential perhaps mediated by increased p21 and p27, drug-induced defects in tumor-initiating cells. When we examined drug-tolerant Du145 cells for the levels of hTERT, which is essential for normal prostate stem/progenitor cells as shown by our recent studies [45], we found that Du145-Paclitaxel and Du145-WP1103 cells, which lacked tumorigenic potential, lost hTERT expression (Fig. 6A). Our previous studies have demonstrated that the clonogenic and tumorigenic potential of Du145 cells largely resides in the CD44^+^ cell fraction [38], [39] and it has recently been shown by one of our groups that paclitaxel conjugated to hyaluronic acid, designed to specifically target the CD44-expressing cells, exhibited potent anti-ovarian cancer effects [49].

The above discussions raised the possibility that perhaps CD44^+^ cells and/or CD44 expression were reduced or ablated in drug-tolerant Du145 cell lines. To test this possibility, we carried out both Western blotting and immunofluorescence experiments. Remarkably, both Du145-Paclitaxel and Du145-WP1103 cultures completely lacked CD44^+^ cells (Fig. 6B–D). Du145-VP16 and Du145-STS cultures also showed reduced CD44 protein levels (Fig. 6B) and numbers of CD44^+^ cells (Fig. 6C–D). By contrast, Du145-WP1102 cells, which retained some tumor-initiating capacity (Table 1), showed similar levels of CD44 protein expression to (Fig. 6B) or slightly more CD44^+^ (many faintly positive) cells (Fig. 6C–D) than parent Du145 cultures. Hence, the extent to which CD44^+^ cells were ablated in drug-tolerant Du145 cultures appeared to correlate well with the level of reduction in their tumorigenic potential. In contrast to CD44, ABCG2 protein (Fig. 6B) or ABCG2^+^ cells (not shown), which constituted ∼1% of total Du145 cells [37], did not show consistent and significant changes in drug-tolerant Du145 cells.

### Requirement of CD44 in Du145 cell tumorigenicity

**Figure 7:**
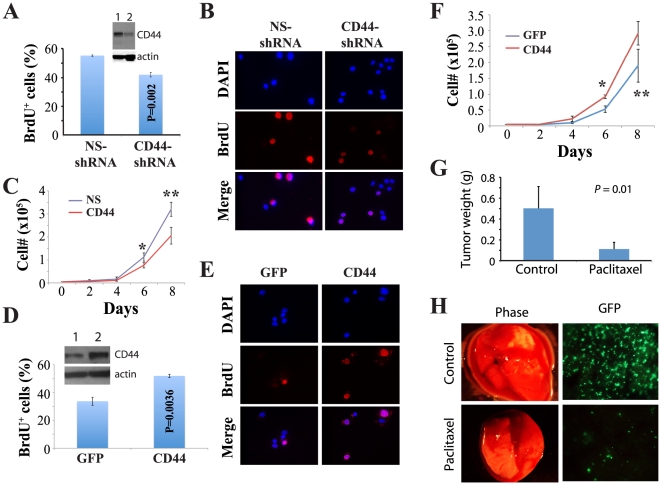
CD44 knockdown and overexpression in Du145 cells inhibits and promotes, respectively, cell proliferation. A–C. CD44 knockdown experiments. Du145 cells infected with the control (NS) or CD44 shRNA lentivectors (MOI 20; 72 h) were plated (10,000/well) in triplicate, pulsed by BrdU for 4 h, and processed for BrdU staining (see Materials & Methods). Shown in (A) is the quantification of BrdU^+^ cells from a total of 500 cells counted for each and in (B) are representative images (×400). Inset, Western blot showing reduced CD44 protein expression in Du145 cells infected with CD44-shRNA (lane 2) compared with the cells infected with NS-shRNA (lane 1). For experiments in C, Du145 cells infected with the control (NS) or CD44 lentivector were plated (5,000/well) in quadruplicate on day 0. At the end of day 2, 4, 6, and 8, cells were dissociated and counted by Trypan blue exclusion. Plotted are the live cell numbers (mean ± SD) as a function of time. *P = 0.019; **P = 0.003 (Student's *t*-test). D–F. CD44 overexpression experiments. Du145-VP16 cells infected with GFP or CD44 retroviral vectors (MOI 20; 72 h) were plated (10,000/well) in triplicate, pulsed by BrdU for 4 h, and processed for BrdU staining. Shown in (D) is the quantification of BrdU^+^ cells from a total of 500 cells counted for each condition and in (E) are representative images (×400). Inset, Western blot showing increased CD44 protein expression in Du145-VP16 cells infected with pBabe-CD44 (lane 2) compared with the cells infected with pBabe-GFP (lane 1). For experiments in F, Du145-VP16 cells infected with the GFP or CD44 retroviral vectors were plated (5,000/well) in quadruplicate on day 0. At the end of day 2, 4, 6, and 8, cells were dissociated and counted by Trypan blue exclusion. Plotted are the live cell numbers (mean ± SD) as a function of time. *P = 0.035; **P = 0.028. G–H. Paclitaxel inhibits PC3 orthotopic tumor growth (G) and metastasis (H). Presented in (G) is the tumor weight (mean ± S.D; n = 5 for each group). Tumor incidence is the same (i.e., 5/5) for both groups. Shown in (H) are representative phase and GFP microphotographs of lung metastases, i.e., GFP^+^ foci.

The above surprising finding that drug-induced reduction/loss of CD44^+^ cells correlates with reduction/loss of tumorigenic potential in Du145 DTCs, is fully consistent with our earlier studies showing that the CD44^+^ PCa cells are more tumorigenic and metastatic than the corresponding CD44^−^ cells [38], [39]. To prospectively determine whether CD44 is causally involved in PCa cell tumorigenicity, we infected parental Du145 cells with a lentiviral vector encoding CD44 shRNA (CD44-shRNA) or a non-silencing shRNA (NS-shRNA). CD44-shRNA reduced CD44 protein (Fig. 7A, inset) and inhibited Du145 cell proliferation as evidenced by reduced % of BrdU^+^ cells (Fig. 7A–B) resulting in reduced live cell numbers (Fig. 7C). When implanted either s.c or orthotopically, the CD44-shRNA infected Du145 cells generated significantly smaller tumors [46].

We then performed the reciprocal gain-of-function experiments by overexpressing CD44 in drug-tolerant Du145 cells. Specifically, we infected the Du145-VP16 cells with pBabe-CD44, which led to increased CD44 expression compared to Du145-VP16 cells infected with pBabe-GFP (Fig. 7D, inset). CD44 re-expression in Du145-VP16 cells enhanced cell proliferation as revealed by BrdU incorporation assays (Fig. 7D–E), resulting in increased live cell numbers (Fig. 7F). Pilot studies indicated that CD44 overexpression in Du145-VP16 slightly increased tumorigenicity (tumor incidence was 1/4 vs. 4/6 in Du145-VP16/pBabe.GFP and Du145-VP16/pBabe.CD44, respectively).

The above results (Fig. 7) suggest that a reduction in CD44^+^ cells is, at least partially, involved in the reduced tumorigenicity of drug-tolerant Du145 cells. These observations also imply that conventional drugs such as etoposide and Taxol may directly target tumor-initiating cells. To further explore this latter point, we employed PC3 cells, which are all CD44^+^ [38], to establish orthotopic tumors in the mouse prostate. After tumors developed for ∼3 weeks, we then performed a therapeutic experiment via i.p (intra-peritoneal) injection of paclitaxel. Consistent with the idea that Taxol may directly target CD44^+^ PCa cells, the intravenously injected paclitaxel greatly inhibited PC3 tumor growth (Fig. 7G) as well as metastasis to the lung (Fig. 7H), pancreas and many other organs (not shown).

### Gene expression changes in drug-tolerant Du145 cells

**Figure 8:**
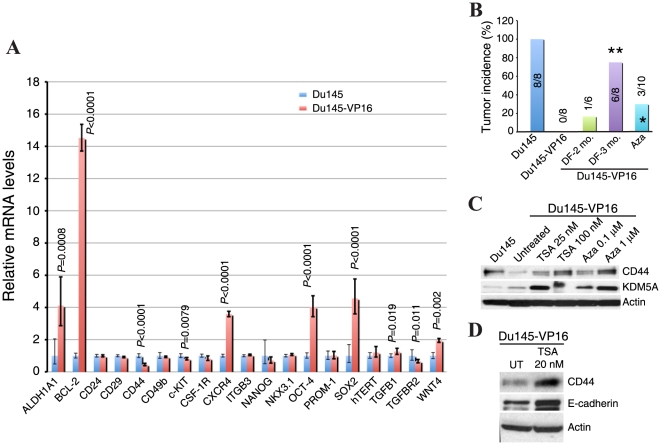
‘Stemness’ gene expression profiles and epigenetic mechanisms in Du145-VP16 cells. A. Du145-VP16 cells show both decreases and increases in stemness gene expression levels. The mRNA levels of the indicated genes in parental Du145 (set at 1, blue bars) and Du145-VP16 (red bars) cells were determined by qPCR. P values were indicated for those genes that showed statistically significant differences. B. Tumor incidence in various types of Du145-VP16 cells implanted (10,000) in NOD/SCID mice. As shown in Table 1, parental Du145 cells showed 100% incidence at 10,000 cells whereas Du145-VP16 cells failed to initiate tumors. When cultured in drug-free (DF) medium for 2 and 3 months, increased tumor incidence was observed (**P<0.01 compared with Du145-VP16). When Du145-VP16 cells treated with Aza (0.1 µM, 72 h) were injected, increased tumor incidence was also observed (*P<0.05 when compared with Du145-VP16 cells). C–D. Du145-VP16 cells treated with the indicated chemicals (72 h) were used in Western blotting analysis of CD44, KDM5A, and E-cadherin. β-Actin was used as loading control.

To further understand what molecular changes might have occurred in drug-tolerant cells, we performed a stem cell SuperArray gene expression analysis of ∼20 ‘stemness’ genes, including ALDH1A1 (the major isoform that mediates the Aldeflour phenotype), BCL-2, CD24, CD29 (integrin β1), CD44, CD49b (integrin α2), c-KIT, CSF-1R, CXCR4, ITGB3 (integrin β3), NANOG, NKX3.1, OCT-4, PROM-1 (CD133), SOX2, hTERT, TGFB1, TGFBR2, and WNT4, in Du145 and Du145-VP16 cells (Fig. 8A). Consistent with our Western blotting and immunostaining results (Fig. 6), CD44 mRNA was significantly lower in Du145-VP16 cells (Fig. 8A). The mRNA levels of c-KIT and TGFBR2 were also significantly reduced (Fig. 8A). The mRNA levels of NANOG and CSF-1R showed a reducing trend but the decrease was not statistically significant (Fig. 8A). Most surprisingly, 6 molecules analyzed, ALDH1A1, BCL-2, CXCR4, OCT-4, SOX2, and WNT4, showed significantly increased mRNA levels in Du145-VP16 cells. These results indicate that the drug-tolerant PCa cells show both decreases and increases in ‘stemness’ genes.

### Evidence for epigenetic mechanisms in generating DTCs

Recently, Sharma et al [50], while modeling the *acute* response of human lung cancer cells to chemotherapeutic drugs, detected a small population of reversibly drug-tolerant cells that possessed an altered chromatin state that involved the KDM5A histone lysine demethylase. To determine whether our DTCs were ‘permanently’ changed by *chronic* drug exposure, we cultured Du145-VP16 and Du145-WP1103 cells in drug-free (DF) medium for 2 months and performed tumor experiments. Such DF-2 month ‘reversion’ experiments revealed that Du145-VP16 cells were still much less tumorigenic and Du145-WP1103 cells still failed to regenerate tumors (Table 1, marked by #; Fig. 8B). However, when we cultured the Du145-VP16 cells in DF medium for 3 months and injected 10,000 such cells into the NOD/SCID mice, we observed 6/8 tumors (Fig. 8B) with mean tumor weight of 0.76±0.24 *g*, of which both tumor incidence and weight were close to those in the parental Du145 cells (Table 1).

The above tumor experiments suggest that the reduced tumorigenicity of drug-tolerant Du145 cells is reversible and may also involve epigenetic mechanisms. To explore this point, we treated Du145-VP16 cells with HDAC (histone deacetylase) inhibitor TSA (trichostatin A) or DNA methyltransferase inhibitor 5′-aza-deoxycytidine (Aza), as we previously described [43]. Both treatments significantly increased the protein levels of CD44, E-cadherin, and KDM5A (Fig. 8C–D). When we injected 10,000 Aza-treated Du145-VP16 cells, we observed 3/10 tumors (Fig. 8B). These results thus implicate epigenetic mechanisms in chronic drug-induced DTC generation.

## Discussion

In this study, we present several surprising and interesting findings related to cancer cell drug resistance (tolerance). The most unexpected finding is that some drug-tolerant cancer cells, contrary to the general assumption, possess much reduced tumor-propagating capacities, the most important functional assay to define the CSC activity [1], [42], [51]. Therefore, both Du145 PCa and DLD1 colon cancer cells chronically selected by multiple drugs demonstrate reduced limiting-dilution tumorigenic potential. In contrast to Du145 and DLD1 cells, 4 of the 6 drug-tolerant UC14 bladder cancer cells show increased tumor-regeneration capacity but 2 drug-tolerant UC14 cell lines also display decreased tumorigenic potential. These observations suggest that DTCs may not necessarily always be endowed with CSC phenotypes or properties; on the contrary, DTCs may actually be depleted of tumor-initiating cells. Our findings are consistent with the emerging evidence that CSCs may have their unique Achilles' heel and *CAN* be preferentially eliminated by certain interventions. For example, glioblastoma CSCs are very sensitive to temozolomide [52] or blocking L1CAM signaling [53]; ovarian CSCs in the side population are sensitive to TNFα [54]; colon CSCs can be efficiently killed by γδ T lymphocytes [55]; and breast CSCs are selectively targeted by salinomycin [56], metformin [57], or conventional chemodrugs [58].

The other surprising and remarkable finding of the present study is that etoposide and taxanes, the two chemotherapy drugs commonly used in the clinical treatment of advanced PCa and some other cancers, preferentially deplete the CD44^+^ Du145 tumor cell population that is known to be enriched in tumor-initiating cells [38], [39]. Our results bear resemblance to recent data by Aulmann et al [58] showing that conventional chemotherapeutic drugs such as docetaxel and doxorubicin can efficiently target the CD44^+^CD24^−^ breast CSCs. In our studies, both paclitaxel and the paclitaxel analog, WP1103, appear to have completely eliminated CD44^+^ Du145 cells and the resultant drug-resistant cells essentially lack tumorigenic potential. VP16 and STS partially eradicate CD44^+^ Du145 cells and the resultant DTCs display only a partial reduction in tumor-initiating capacity. In contrast, WP1102, another paclitaxel-related compound, does not significantly affect the abundance of CD44^+^ cells, and consequently, Du145-WP1102 cells retain notable tumorigenic ability. It is possible that WP1102, which is structurally similar to WP1103, is not as potent as WP1103 because the ‘optimal’ concentration of WP1102 chosen for the study was lower than that of WP1103. Regardless, the extent to which CD44^+^ Du145 cells are depleted correlates well with the reduction in tumorigenicity of DTCs. Importantly, prospective knockdown of CD44 in Du145 cells greatly inhibits Du145 cell proliferation and tumor regeneration. Knockdown of CD44 in several other PCa models including PC3, LAPC4, and LAPC9 also significantly inhibits tumor development and/or metastasis [46]. By contrast, overexpression of CD44 in drug-tolerant Du145 cells enhances cell proliferation and slightly restores tumorigenicity. These observations not only corroborate our earlier contention that the CD44^+^ PCa cell population harbors tumor-initiating cells, but also establish requirements and the importance of CD44 and CD44^+^ cells in PCa development, consistent with other studies implicating critical/functional roles of CD44 in various CSCs and tumor systems including leukemic stem cells [59], [60], colon CSCs [10], gastric CSCs [18], and ovarian tumors [61]. It should be pointed out that in drug-tolerant DLD1 cells, we did not observe a significant reduction in CD44 protein levels or CD44^+^ cells (Yan et al., unpublished observations), suggesting that drug-induced CD44 depletion may be cell type-dependent.

The third surprising finding of great interest is that drug-tolerant Du145 cells show both decreases and increases in many stem cell-associated molecules, implicating epigenetic mechanisms in chronic drug selection-engendered DTCs. Indeed, although drug-tolerant Du145-VP16 cells completely lack tumorigenicity, culturing the same cells in DF medium for 2 or 3 months, in a time-dependent manner, restores their tumor-initiating capacity (Fig. 8B). Furthermore, treatment of Du145-VP16 cells with 5′-aza-deoxycytidine for as short as 72 h significantly increases their tumorigenic potential. At the molecular level, treatment of DTCs with TSA or Aza increases the protein levels of CD44, E-cadherin, and KDM5A, indicating strongly that chronic drug exposure has altered the epigenetic landscape and chromatin state in Du145 cells. These observations, which are remarkably similar to the altered chromatin state also involving KDM5A observed in drug-tolerant lung cancer cells during their acute response to chemotherapeutic drugs and tyrosine kinase inhibitors [50], suggest that the loss of tumorigenicity in chronic drug-selected Du145 cells is ‘transient’ and reversible. These results further bear resemblance to the emerging evidence that cancer cells can cyclically lose and regain drug-resistant CSC features and properties [62]. Finally, our findings provide some mechanistic insight on the current metronomic chemotherapy and imply that continuous administration of low doses of chemodrugs can keep drug-tolerant cancer cells at bay and prevent them from regenerating tumors. Because the drug-tolerant Du145 cells have undergone multiple epigenetic changes (Fig. 8), understandably, CD44 overexpression alone can only partially restore the tumorigenic potential of Du145-VP16 cells and CD44 knockdown in parent Du145 cells, despite inhibiting cell proliferation and tumor regeneration, does not render cells resistant to drugs (Chen et al., unpublished observations).

How should we reconcile the current findings with other studies showing that drug-resistant cancer cells are enriched in stem-like cancer cells [31], [63], [64]? In most drug-resistance studies, cancer cells are exposed acutely to therapeutics for a short period of time, generally up to 2 weeks. In contrast, we purposely wish to recapitulate the metronomic chemotherapy [48] currently used in the clinic by CHRONICALLY exposing cancer cells to drugs for months. Presumably, the difference in drug-selection protocols (i.e., the dose of drugs and duration of exposure) has led to more or less tumorigenic cancer cells. It is interesting to note that etoposide and taxanes are among the few chemotherapy drugs that have demonstrated some clinical efficacy for advanced PCa patients. It is currently unclear how etoposide and paclitaxel, which possess quite different modes of action, preferentially ablate the CD44^+^ Du145 cells. If the clinical efficacy of these two drugs is associated with their ability to target the CD44^+^ PCa cells and if CSCs, as hypothesized, represent the cells at the root of tumor maintenance, progression, recurrence, and metastasis, why cannot etoposide and paclitaxel completely ablate the tumors and cure the patients? One possibility is related to inefficient delivery of drugs such that they cannot reach all CD44^+^ cells in the tumor. Another possibility is that PCa cells may possess some plasticity and CD44^−^ PCa might revert to CD44^+^ PCa cells. Indeed, CD44^−^ Du145-VP16 cells, after culturing in DF medium for 3 months, contain nearly as many CD44^+^ cells as in the parent Du145 cultures (unpublished observations). Future studies on DTCs should shed important light on the cellular, molecular, and epigenetic mechanisms of cancer cell drug resistance.

## Supporting Information

Table S1 Reduced tumorigenic potential in drug-tolerant DLD1 cells.
(DOC)

Table S2 Drug-tolerant UC14 cells demonstrate drug-dependent changes in tumorigenicity.
(DOC)
