# Supplementary material for: Drug-Tolerant Cancer Cells Show Reduced Tumor-Initiating Capacity: Depletion of CD44+ Cells and Evidence for Epigenetic Mechanisms
Source: PLoS One. 2011 Sep 15;6(9):e24397. doi: 10.1371/journal.pone.0024397 (PMC3174165; doi:10.1371/journal.pone.0024397)
Supplement: Table S1 — Reduced tumorigenic potential in drug-tolerant DLD1 cells. (DOC) [file pone.0024397.s001.doc]

**Table S1. Reduced tumorigenic potential in drug-tolerant DLD1 cells**

| Cell line | Cell# | Tumor incidence (%) | Termination (days) | Weights (g) |
| --- | --- | --- | --- | --- |
| DLD1  DLD1- VP16  DLD1-  Paclitaxel  DLD1- WP1102  DLD1- WP1103 | 1000  10000  100000  1000  10000  100000  1000  10000  100000  1000  10000  1000  10000 | 8/8 (100)  8/8 (100)  6/6 (100)  7/8 (87.5)  7/8 (87.5)  6/6 (100)  8/8 (100)  6/8 (75)  6/6 (100)  8/8 (100)  8/8 (100)  8/8 (100)  8/8 (100) | 45  30  30  45  30  30  45  30  30  45  31  45  31 | 0.58 ± 0.22 (0.28-1.03)  0.60 ± 0.25 (0.31-0.97)  0.88 ± 0.18 (0.75-1.24)  0.09 ± 0.11 (0.01-0.31)**  0.08 ± 0.07 (0.01-0.21)**  0.41 ± 0.10 (0.26-0.56)**  0.40 ± 0.22 (0.13-0.80)  0.34 ± 0.15 (0.15-0.54)*  0.65 ± 0.17 (0.49-0.87)*  0.36 ± 0.21 (0.11-0.59)*  0.37 ± 0.18 (0.07-0.60)*  0.46 ± 0.15 (0.21-0.66)  0.28 ± 0.18 (0.04-0.55)** |

Parental or drug-tolerant DLD1 colon cancer cells were implanted, in 50% Matrigel, at various cell numbers s.c in NOD/SCID mice. Presented are tumor incidence (% tumor development), termination time (days since tumor cell injection when animals were sacrificed), and tumor weights (mean ± S.D; ranges in parentheses). *P< 0.05 and **p<0.01, compared with the parental DLD-1 cells of the same numbers.
