# Supplementary material for: Drug-Tolerant Cancer Cells Show Reduced Tumor-Initiating Capacity: Depletion of CD44+ Cells and Evidence for Epigenetic Mechanisms
Source: PLoS One. 2011 Sep 15;6(9):e24397. doi: 10.1371/journal.pone.0024397 (PMC3174165; doi:10.1371/journal.pone.0024397)
Supplement: Table S2 — Drug-tolerant UC14 cells demonstrate drug-dependent changes in tumorigenicity. (DOC) [file pone.0024397.s002.doc]

**Table S2. Drug-tolerant UC14 cells demonstrate drug-dependent changes in tumorigenicity**

| **Cell line^a^** | **Cell#** | **Tumor incidence^b^ (%)** | **Latency^c^ (days)** | **Termination^c^ (days)** | **Tumor weight^d^**  **(grams)** |
| --- | --- | --- | --- | --- | --- |
| UC14  UC14- Paclitaxel  UC14- STS  UC14- Dox  UC14- VP16  UC14- WP1102  UC14- WP1103 | 1,000  10,000  100,000  1,000  10,000  100,000  1,000  10,000  100,000  1,000  10,000  100,000  1,000  10,000  100,000  1,000  10,000  100,000  1,000  10,000  100,000 | 6/8 (75.0)  8/8 (100)  5/6 (83.3)  8/8 (100)  8/8 (100)  5/6 (83.3)  5/8 (62.5)  8/8 (100)  6/6 (100)  4/8 (50)  8/8 (100)  6/6 (100)  0/8 (0)^Δ^  6/8 (75)  5/6 (83.3)  4/8 (50)  5/6 (83.3)  6/6 (100)  8/8 (100)  7/8 (87.5)  6/6 (100) | 23  15  13  20^#^  15  13  26  18  18  N.D  22^##^  13  20^#^  13  27  19  13  22  18  13 | 38  29  24  38  29  24  38  29  24  38  29  24  29  24  38  29  24  38  29  24 | 0.15±0.16 (0.01-0.41)  0.32±0.10 (0.11-0.46)  0.23±0.17 (0.09-0.43)  0.66±0.37 (0.18-1.16)**  0.42±0.15 (0.15-0.60)  0.38±0.13 (0.20-0.54)  0.08±0.06 (0.03-0.16)  0.33±0.27 (0.09-0.81)  0.53±0.16 (0.30-0.76)**  0.01±0.01 (0.01-0.02)*  0.12±0.09 (0.02-0.27)**  0.27±0.13 (0.16-0.45)  0.04±0.04 (0.01-0.09)**  0.08±0.02 (0.05-0.09)  0.17±0.18 (0.01-0.35)  0.28±0.25 (0.02-0.61)  0.42±0.14 (0.21-0.59)*  0.34±0.37 (0.01-1.15)  0.57±0.22 (0.24-0.96)*  0.48±0.09 (0.39-0.64)* |

^a^Parental UC14 or drug-tolerant UC14 cells were implanted s.c, in 50% Matrigel, at the numbers indicated, in NOD/SCID mice.

^b^Tumor incidence (% of tumor development/injections). Δ, P< 0.05, compared with UC14 at the same cell dose.

^c^Tumor latency refers to mean time in days from injection to when tumors were first palpated. Δ, P< 0.05, and ΔΔ, P<0.01, when compared with UC14 at the same cell doses. All animals injected with the same number of tumor cells were terminated at the same time.

^d^Mean ± S.D (ranges in parentheses). *, P< 0.05, and **, P<0.01, compared with UC14 at the same cell doses.

For statistical analyses, Fisher’s Exact Test was used to compare tumor incidence and latency and Student *t*-test was used to compare tumor weights and latencies.
